# Single-cell ligand–receptor profiling reveals an immunotherapy-responsive subtype and prognostic signature in triple-negative breast cancer

**DOI:** 10.3389/fimmu.2025.1590951

**Published:** 2025-06-10

**Authors:** Chuanzhi Chen, Jiahui Qi, Weichao Lin, Chunlan Fu, Xin Jin

**Affiliations:** ^1^ Department of Thyroid Surgery, National Key Clinical Specialty (General Surgery), The First Affiliated Hospital of Wenzhou Medical University, Wenzhou, Zhejiang, China; ^2^ Institute of Aging, Key Laboratory of Alzheimer’s Disease of Zhejiang Province, Wenzhou Medical University, Wenzhou, Zhejiang, China; ^3^ Department of Hematology and Breast Surgery, Zhuji Affiliated Hospital of Wenzhou Medical University, Zhuji, Zhejiang, China

**Keywords:** tumor microenvironment, ligand-receptor interactions, triple-negative breast cancer, prognostic model, immunotherapy, immune checkpoints

## Abstract

**Background:**

Triple-negative breast cancer (TNBC) is an aggressive form of cancer that lacks specific targeted therapies. Although ligand–receptor (LR) interactions play a crucial role in intercellular communication and contribute to tumor heterogeneity, their molecular details and potential as prognostic or predictive markers in TNBC have not been thoroughly investigated.

**Methods:**

We analyzed single-cell RNA sequencing data to categorize TNBC into 12 subgroups and 10 distinct cell types. From this dataset, we identified LR pairs that exhibited significant intercellular crosstalk and evaluated their prognostic relevance in a METABRIC TNBC cohort (n = 298). Through consensus clustering of these LR pairs, two molecular subtypes were defined. Key LR genes were then selected using Lasso regression and stepwise multivariate analysis to build an LR-based prognostic scoring system (LR.score), which was validated using both the METABRIC and GSE58812 datasets (n = 107). Additionally, we performed siRNA-mediated knockdown of the CXCL9/CXCR3 axis in MDA-MB-231 cells, confirming the knockdown via RT-qPCR and Western blot. The functional impact was assessed through proliferation, colony formation, and wound healing assays.

**Results:**

One subtype (Clust1) demonstrated strong immune cell infiltration, higher immune scores, and enrichment in pathways such as epithelial–mesenchymal transition, angiogenesis, and KRAS signaling—indicative of a basal-like, immune-active phenotype. Among the LR pairs, the CXCL9–CXCR3 axis was identified as a key factor in immune cell recruitment and anti-tumor responses. Functionally, silencing the CXCL9/CXCR3 axis significantly diminished the proliferation, colony formation, and migratory capabilities of MDA-MB-231 cells. Moreover, a higher LR.score was correlated with poorer overall survival (HR = 1.69, 95% CI = 1.12–2.56, P < 0.05) and reduced response to immune checkpoint inhibitors (ICIs), while patients with lower LR.score showed increased sensitivity to ICIs, particularly in anti–PD-L1 cohorts.

**Conclusion:**

The LR.score serves as an independent prognostic factor and a reliable predictor of immunotherapy response in TNBC. Targeting crucial LR interactions, especially the CXCL9–CXCR3 axis, may enhance immunotherapeutic efficacy and refine prognostic evaluations, paving the way for improved treatment strategies in TNBC.

## 1 Introduction

Triple-negative breast cancer (TNBC) remains one of the most challenging and aggressive breast cancer subtypes, primarily due to the absence of estrogen, progesterone, and HER2 receptors. This inherent lack of recognized therapeutic targets severely limits treatment options and highlights the urgency of identifying novel biomarkers and therapeutic strategies. Central to understanding TNBC progression and drug resistance is the tumor microenvironment (TME), a complex ecosystem composed of malignant cells, immune cells, and non-immune stromal components. These heterogeneous cell populations communicate through ligand-receptor (LR) interactions—molecular events that govern key processes such as tumor initiation, progression, and immune evasion (1, 2).

While traditional bulk RNA sequencing (bulk RNA-seq) has been invaluable in providing global gene expression profiles, it obscures the considerable heterogeneity inherent to tumors by averaging signals across millions of cells. In contrast, single-cell RNA sequencing (scRNA-seq) enables high-resolution analyses of individual cells, capturing their distinct transcriptional states and shedding light on heterogeneous cell populations within the TME. This refined approach is especially relevant for TNBC, where diverse subclones and tumor-associated immune or stromal cells engage in dynamic crosstalk, influencing tumor behavior and therapy outcomes (3, 4).

Recent studies underscore that immune heterogeneity within the TME is crucial for predicting therapy response. Ligand-receptor (LR) crosstalk, in particular, has emerged as a key regulator of TNBC subtypes and a potential target for intervention (5). Single-cell approaches offer comprehensive insights into immune cell distributions and functions, elucidating immune evasion mechanisms that TNBC tumors employ (6). Notably, immune checkpoint regulators—such as PD-L1—and their interactions with T cells within the TME play a pivotal role in immune suppression and therapy resistance (7).

Building upon these findings, we leveraged scRNA-seq data to dissect the TME in TNBC, focusing on LR interactions that may drive clinical heterogeneity. By integrating single-cell transcriptomic data with large-scale genomic and clinical datasets, including METABRIC and GEO, we aimed to identify clinically relevant LR pairs, define molecular subtypes according to LR expression profiles, and assess their prognostic impact and relationship to immunotherapy response. This comprehensive analysis provides novel perspectives on TNBC pathobiology and unveils potential strategies for more precise prognostic assessment and targeted therapeutic interventions.

## 2 Material and methods

### 2.1 Data source and pre-processing

**Figure 1:**
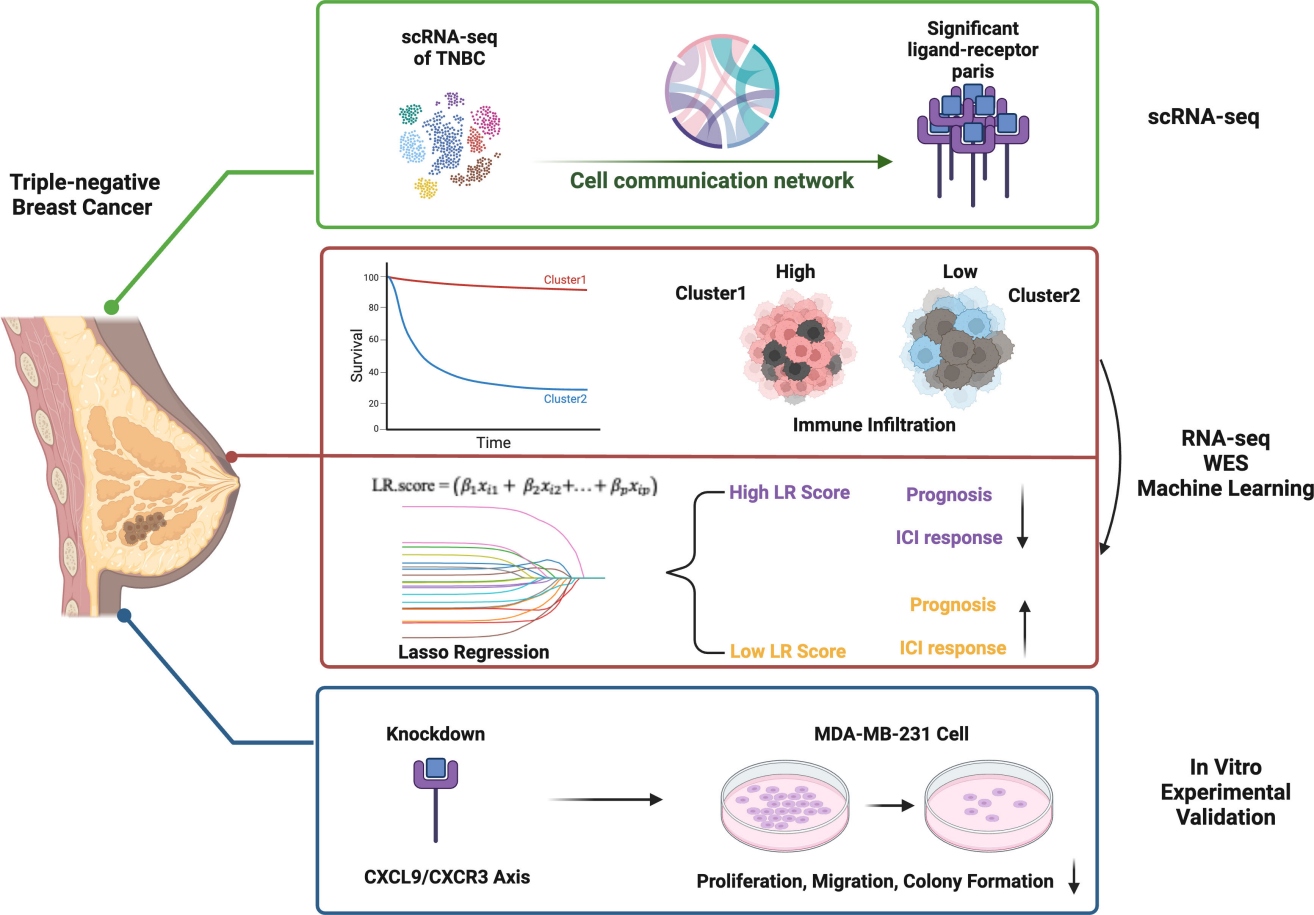
Graphic abstract.

We obtained single-cell RNA sequencing (scRNA-seq) data for this study from the NCBI GEO repository under the accession number GSE176078. This dataset comprises nine individual samples. To ensure data integrity, we applied rigorous quality control measures: cells with a high mitochondrial gene fraction (≥25% for tumor libraries) or a low count of detected genes (<300) were deemed low-quality and excluded. After filtering, 38,007 high-quality cells were retained (**Supplementary Table S1**). Additionally, we retrieved the METABRIC dataset (8, 9) accessed through the cBioPortal (http://cbioportal.org/). Within this dataset, we identified 318 triple-negative breast cancer (TNBC) samples, of which 298 provided both gene expression profiles and genomic variation data. Additionally, we retrieved microarray data (GSE58812) from GEO. Following probe annotation and conversion to gene symbols, we removed normal tissue samples, excluded those lacking clinical follow-up or overall survival (OS) data, and obtained a final cohort of 107 tumor samples, each with expression data for 16,416 genes. For METABRIC and GSE58812 we performed robust-multi-array average (RMA) normalization and then log_2_-transformed, gene-wise Z-scaled expression values within each cohort. Because all downstream modeling (screening, LASSO training and external validation) was carried out separately inside each dataset, no additional cross-platform batch-correction step was required. These datasets, encompassing single-cell and bulk-level transcriptomic information, served as the foundation for subsequent molecular and clinical analyses. The study outline is illustrated in **Figure 1**.

### 2.2 Patients stratification and survival analysis

For each ligand–receptor (LR) pair, we classified patients as “high” if the sum of the two genes expression reached or exceeded the median of all samples combined LR expression. Otherwise, patients were classified as “low.” The combined LR expression was defined as the sum of the expression levels of both genes in a pair. We evaluated overall survival using the “Survival” package in R (version 4.2.3), determining statistical significance via log-rank tests and deriving hazard ratios (HR) from Cox regression models. Survival analyses were performed independently for each cohort, their p-values were then combined with the Edgington method using the “sump” function in the “metap” package (version 1.4). Finally, Storey’s method (10) from the “qvalue” package (version 2.18.0) was applied for multiple testing corrections. Prognosis-related LR pairs were defined by two criteria across all cohorts: (1) Storey’s q-value < 0.1, and (2) a consistent HR either > 1 or < 1.

### 2.3 scRNA-seq data analysis

We conducted all single-cell analyses in R (version 4.2.3) using the Seurat package (version 4.3.0). As part of our quality control (QC), we excluded cells with either (i) an elevated fraction of mitochondrial genes (≥25% for tumor libraries), which often indicates stressed or dying cells, or (ii) a low gene count (<300), which may reflect poor capture efficiency. We also examined and visualized the correlation between the percentage of mitochondrial genes and the total mRNA reads, including how the number of detected genes relates to overall sequencing depth.

After normalizing the data, we identified highly variable genes (HVGs) by controlling for their average expression and dispersion. Next, principal component analysis (PCA) was performed using these HVGs as input, and the jackStraw function guided the selection of significant principal components (PCs). When merging data from different samples, we re-identified HVGs and applied canonical correlation analysis (CCA) to remove batch effects. The cells were then embedded into a two-dimensional space via t-distributed stochastic neighbor embedding (t-SNE), enabling clear visualization of their transcriptional landscapes. Using a resolution of 0.2, we employed FindClusters to group cells into 12 distinct clusters (labeled 0–11). To further characterize the transcriptional profiles of each cluster, we used FindAllMarkers to detect differentially expressed genes (DEGs) between each cluster and all other cells. This workflow yielded a comprehensive view of cellular diversity and underlying gene expression patterns in our single-cell dataset. For cell annotation, We obtained classic markers for defining cell subsets from previous studies (11) and performed manual annotation of the cell clusters based on the expression of these markers.

### 2.4 Cell–cell communication and ligand–receptor pair analysis

We investigated intercellular communication using CellPhoneDB (12),a curated database of known ligands, receptors, and their interactions. As part of this analysis, we annotated membrane-bound, secreted, and peripheral proteins across different cell clusters. We then performed a permutation test to assess the significance of each putative interaction, drawing on the normalized cell expression matrix to calculate mean interaction values. For each pair of cell clusters, we identified all ligand–receptor interactions with a nominal p-value below 0.05. To focus on biologically relevant associations, we only retained interactions where at least one partner was a receptor (according to the CellPhoneDB annotations), thereby excluding receptor–receptor or other ambiguous pairs lacking a clear receptor component. This approach enabled us to construct a detailed communication network that reveals how distinct cell populations within the tumor microenvironment potentially coordinate key processes such as proliferation, immune response, and disease progression.

### 2.5 Samples clustering through consensus clustering

We employed the “ConsensusClusterPlus” package in R (13) to construct a consensus (consistency) matrix and cluster our samples, thereby identifying molecular subtypes. First, we focused on significantly correlated ligand-receptor pairs, selecting those with a Pearson correlation coefficient above 0.4 (*P* < 0.01). These high-correlation pairs were then subjected to consensus clustering to determine molecular subtypes. For the clustering itself, we used the Pearson metric and the “pam” algorithm, running 500 bootstrap replications. In each bootstrap iteration, we randomly sampled 80% of the patients from the training set. We explored solutions ranging from 2 to 10 clusters. The most stable clustering outcome was chosen based on inspection of the consensus matrix and the consensus cumulative distribution function (CDF), ensuring that the final partitioning was both reproducible and robust.

### 2.6 Development of the LR.score prognostic model

We developed a personalized prognostic model by selecting ligand-receptor (LR) pairs that displayed a significant impact on patient outcomes and incorporating them into a penalized Cox regression. Specifically, we employed L1-penalized Least Absolute Shrinkage and Selection Operator (LASSO) regression via the “glmnet” package in R. The optimal λ value—governing the penalty intensity—was identified through ten-fold cross-validation, whereby the dataset was split into ten subsets and each one used as a validation set in turn. The criterion for choosing λ was minimizing the partial likelihood deviance, thus retaining the most predictive variables and shrinking the coefficients of less relevant ones to zero, thereby striking a balance between performance and overfitting avoidance.

Following variable selection through LASSO, we refined our model using a stepwise multivariate regression approach based on the Akaike Information Criterion (AIC) via the “stepAIC” function in the “MASS” package. By iteratively removing variables to reduce the AIC, we achieved an optimal compromise between model complexity and fit. Ultimately, 6 LR pairs remained stable across multiple models and consistently contributed to survival prediction, forming the basis of the LR.score. The patient-specific risk score was computed using the following formula:


$$\begin{array}{c} LR.score\;=\;=\;LR.score\;\\ =\;-0.431\;*\;WNT1\_ROR2\;-\;0.081\;*\;CXCR3\_CXCL9\;\\ -\;0.296\;*\;FGFR2\_CD83\;+\;0.467\;*\;TIMP1\_FGFR2\;\\ +\;0.426\;*\;NGFR\_IL2\;-\;0.051\;*\;HLA-A\_KIR3DL1. \end{array}$$

### 2.7 Functional annotations and immune infiltration analysis

To investigate the distinct gene expression profiles across various molecular subtypes, we conducted Gene Set Enrichment Analysis (GSEA v4.0) (14) using the Hallmark database (15). We considered results statistically significant if the normalized p-value was below 0.01 and the False Discovery Rate (FDR) was under 0.05. For further insight into differentially expressed genes in each subtype, we employed the “clusterProfiler” package [30] to perform Kyoto Encyclopedia of Genes and Genomes (KEGG) pathway analyses, thereby identifying key biological pathways affected. Functional annotation was also carried out using the clusterProfiler package.

To assess immune infiltration in triple-negative breast cancer, we utilized the CIBERSORT (16) algorithm (https://cibersort.stanford.edu/) to estimate the relative abundance of 22 immune cell types. Additionally, we applied the ESTIMATE software to determine the proportion of immune cells in the tumor microenvironment, providing a deeper understanding of the immune landscape associated with each subtype.

#### 2.7.1 Cell culture and knockdown of CXCL9 and CXCR3 genes

MDA-MB-231 cells were obtained from ATCC, USA and cultured in Dulbecco’s Modified Eagle Medium (DMEM) supplemented with 10% fetal bovine serum (FBS) and 1% penicillin-streptomycin at 37°C in a humidified incubator with 5% CO_2_. The knockdown of CXCL9 and CXCR3 genes was achieved using small interfering RNAs (siRNAs) specific to these genes, purchased from Thermo Fisher Scientific with catalog numbers s141037 and s64088. Cells were seeded in 6-well plates at a density of 2 × 10^5^ cells per well and incubated overnight until they reached approximately 60–70% confluence. siRNAs were diluted in Opti-MEM™ Reduced Serum Medium (Thermo Fisher Scientific) to a final concentration of 50 nM and mixed with Lipofectamine™ RNAiMAX Transfection Reagent (Thermo Fisher Scientific) following the manufacturer’s instructions to form siRNA-transfection reagent complexes. These complexes were then added dropwise to the cells in serum-free DMEM. After 6 hours of incubation, the medium was replaced with complete DMEM containing 10% FBS.

To confirm knockdown efficiency, total RNA was extracted from transfected cells 48 hours post-transfection using the TRIzol™ Reagent (Thermo Fisher Scientific). cDNA was synthesized using the High-Capacity cDNA Reverse Transcription Kit (Thermo Fisher Scientific), and quantitative real-time PCR (RT-qPCR) was performed using PowerUp™ SYBR™ Green Master Mix (Thermo Fisher Scientific) with gene-specific primers for CXCL9 and CXCR3, with GAPDH as an internal control for normalization The ΔCt method was used to calculate the relative expression levels of CXCL9 and CXCR3. The cycle threshold (Ct) values of the target genes were normalized to the Ct value of GAPDH using the formula ΔCt = Ct(target gene) - Ct(GAPDH). Following primer pairs were used for amplification purpose:

GAPDH-F 5’-ACCCACTCCTCCACCTTTGAC-3’,
GAPDH-R 5’-CTGTTGCTGTAGCCAAATTCG-3’
CXCL9-F: 5’-CTGTTCCTGCATCAGCACCAAC-3’
CXCL9-R: 5’-TGAACTCCATTCTTCAGTGTAGCA-3’
CXCR3-F: 5’-ACGAGAGTGACTCGTGCTGTAC-3’
CXCR3-R: 5’-GCAGAAAGAGGAGGCTGTAGAG-3’

Additionally, Western blotting was performed to validate the protein-level knockdown of CXCL9 and CXCR3. Briefly, total protein was extracted from transfected cells 48 hours post-transfection using RIPA buffer (Thermo Fisher Scientific) supplemented with protease and phosphatase inhibitor cocktail (Thermo Fisher Scientific). Protein concentrations were measured using the Pierce™ BCA Protein Assay Kit (Thermo Fisher Scientific). Equal amounts of protein (20–30 µg per lane) were separated by SDS-PAGE on 10% polyacrylamide gels and transferred onto PVDF membranes (Thermo Fisher Scientific). The membranes were blocked with 5% non-fat dry milk in TBS-T (Tris-buffered saline with 0.1% Tween-20) for 1 hour at room temperature and incubated overnight at 4°C with primary antibodies specific to CXCL9 (Cat # PA5-34743, Thermo Fisher Scientific) and CXCR3 (Cat # PA5-88164, Thermo Fisher Scientific), as well as GAPDH (loading control). The membranes were washed three times with TBS-T and incubated with HRP-conjugated secondary antibodies (Thermo Fisher Scientific) for 1 hour at room temperature. Protein bands were visualized using the Pierce™ ECL Western Blotting Substrate (Thermo Fisher Scientific) and imaged using a chemiluminescent imaging system. Densitometric analysis of the bands was performed using ImageJ software to quantify the knockdown efficiency of CXCL9 and CXCR3 at the protein level.

#### 2.7.2 Cell proliferation assay

Cell proliferation was assessed using the CellTiter 96^®^ AQueous One Solution Cell Proliferation Assay (MTS Assay) kit (Promega). Transfected cells were seeded into 96-well plates at a density of 2 × 10³ cells per well in triplicates and incubated in DMEM supplemented with 10% FBS at 37°C in a humidified 5% CO_2_ incubator. At 24-, 48-, and 72-hours post-seeding, 20 µL of the MTS reagent was added to each well, and the plate was incubated for 2 hours at 37°C. Absorbance was measured at 490 nm using a microplate reader to assess cell viability, which reflects cell proliferation.

#### 2.7.3 Colony formation assay

The colony formation assay was used to assess the clonogenic ability of the cells. Transfected cells were seeded into 6-well plates at a low density of 500 cells per well and incubated for 10–14 days in DMEM containing 10% FBS until visible colonies formed. The medium was replaced every 3–4 days. After the incubation period, the cells were washed with phosphate-buffered saline (PBS), fixed with 4% paraformaldehyde for 15 minutes, and stained with 0.1% crystal violet for 30 minutes. Excess stain was washed off with distilled water, and the plates were air-dried. Colonies consisting of ≥50 cells were counted manually under a microscope, and the data were normalized to the control group.

#### 2.7.4 Wound healing assay

The wound healing assay was performed to evaluate the migration capability of transfected MDA-MB-231 cells. Cells were seeded into 6-well plates and grown to 90–100% confluence in DMEM supplemented with 10% FBS. A sterile 200 µL pipette tip was used to create a straight scratch (wound) across the cell monolayer. The wells were washed twice with PBS to remove floating cells and debris, and the cells were then cultured in serum-free DMEM. Images of the wound area were captured at 0 and 24 hours using an inverted microscope. The wound area was measured using ImageJ software, and the percentage of wound closure was calculated as follows:


$$\begin{array}{c} Wound\;Closure\;\left(\%\right)\;\\ =\;[(Initial\;Wound\;Area\;\\ -\;Final\;Wound\;Area)/Initial\;Wound\;Area]\;\times \;100. \end{array}$$

## 3 Results

### 3.1 Screening of LR pairs associated with patient prognosis

**Figure 2:**
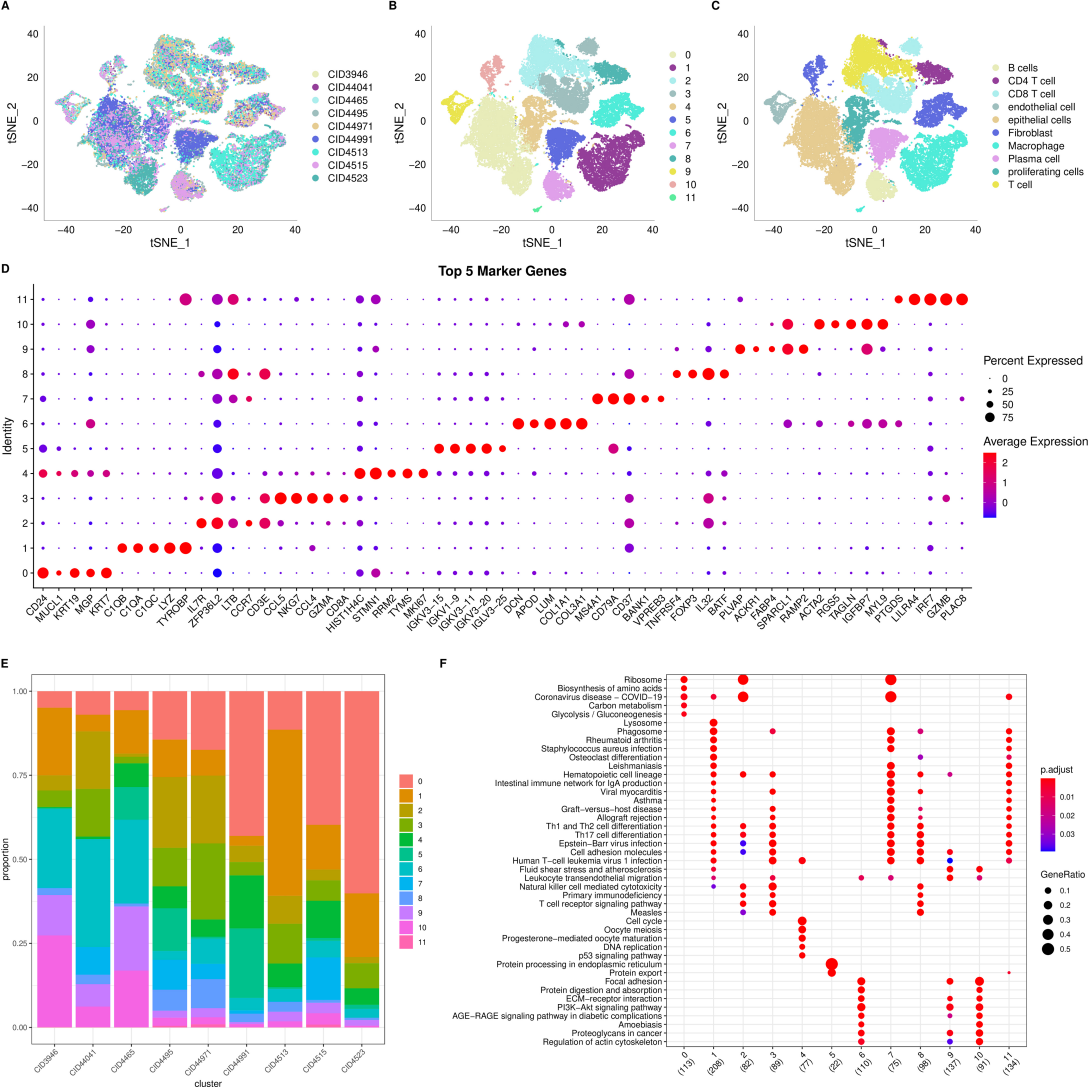
Single cell RNA seq analysis. **(A)** t-SNE distribution plot for 9 samples; **(B)** t-SNE distribution plot for 12 subgroups; **(C)** Annotated t-SNE plot showing cell distribution; **(D)** Dot plot showing the expression of the top 5 marker genes for each of the 12 subgroups; **(E)** Distribution of the 12 subgroups across the 9 samples; **(F)** Dot plot showing the results of KEGG enrichment analysis for the 12 subgroups.

We began by filtering the single-cell RNA-seq (scRNA-seq) data to include only genes expressed in at least three cells and cells expressing a minimum of 250 genes, yielding 38,582 cells initially. We then assessed mitochondrial and rRNA content, ensuring that each cell had 100 to 6,000 genes expressed, <20% mitochondrial content, and at least 100 UMIs, resulting in a final set of 38,007 cells. Using the first 30 principal components and a resolution of 0.2, we identified 12 subgroups. The t-SNE plot for the nine samples (**Figure 2A**) demonstrates successful mixing of samples, indicating effective batch correction. We then manually annotated the cells (**Supplementary Figure S1**, **Supplementary Table S2**), identifying 10 cell types in total. **Figures 2B, C** display the t-SNE plots before and after annotation, respectively.

To characterize the subgroups further, we used the “FindAllMarkers” function to identify differentially expressed genes (DEGs) for each of the 12 clusters. We set the following thresholds: log fold change > 0.5, a minimum proportion of differentially expressed genes > 0.35, and adjusted p-value < 0.05. The top five DEGs for each cluster are visualized in **Figure 2D**, and the complete DEGs are listed in **Supplementary Table S3**. We also examined the distribution of the 12 clusters across the nine samples (**Figure 2E**) and performed KEGG pathway annotation for the DEGs in each cluster. Notably, clusters 2 and 7 are enriched in “Ribosome,” while cluster 5 is enriched in “Protein processing in the endoplasmic reticulum” (**Figure 2F**).

### 3.2 Complex cell communication networks in the tumor microenvironment

**Figure 3:**
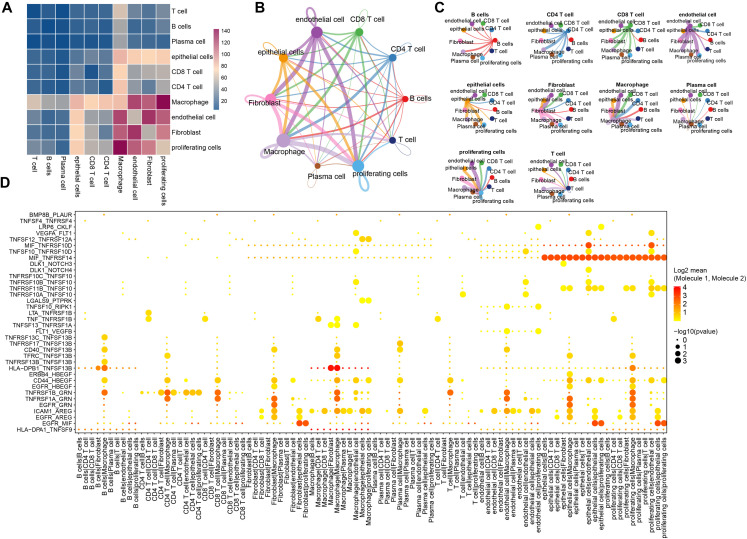
The ligands-receptors interactions in different cells. **(A)** Depiction of ligand-receptor interactions between different cell populations as determined by CellphoneDB; **(B)** Network diagram from CellChat illustrating the number of significant interaction events between different cell populations, the thickness of each line represents the connection strength between each cell; **(C)** Another networks representation showing the count of significant interaction events between various cell populations, the thickness of each line represents the connection strength between each cell; **(D)** A broad overview of selected statistically significant interactions between cell types, as identified by CellphoneDB.

We employed the CellPhoneDB framework to investigate potential interactions among different cell types within the triple-negative breast cancer tumor microenvironment. As shown in **Figure 3A**, multiple interactions are evident, with macrophages exhibiting particularly strong communication with endothelial cells and proliferating cells. An overview of the interaction networks among the ten cell subgroups is provided in **Figure 3B**, highlighting extensive interactions both within and between these subgroups. In this network, thicker lines signify a greater number of significant ligand–receptor pairs between subgroups, while larger nodes indicate a higher abundance of these pairs. Notably, endothelial cells, macrophages, and fibroblasts emerged as hubs of communication, displaying robust interactions both among themselves and with other subgroups (**Figure 3C**).

To further elucidate the significance of these interactions, we focused on genes associated with tumor-related pathways such as Hedgehog, Notch, TGFβ, WNT, and EGFR signaling. This analysis revealed numerous interactions involving MIF_TNFRSF14 in both endothelial and proliferating cell subgroups, as well as notable HLA-DPB1_TNFSF13B interactions between B cells and fibroblasts, macrophages and fibroblasts, and even within macrophages (**Figure 3D**). A detailed summary of these findings is provided in **Supplementary Table S4**.

### 3.3 Molecular subtyping based on ligand-receptor pairs

In our cell-to-cell communication analysis, we observed significant differences in the expression of receptors and ligands, receptor-ligand interaction strength, the number of receptor-ligand pairs, and the types of receptor-ligand pairs across different cell types. These differences may lead to the activation or inhibition of various pathways, ultimately resulting in tumor development, progression, and drug resistance. Therefore, we extracted significantly interacting ligand-receptor pairs across different cell types and, based on the Pearson’s correlation coefficients of receptor and ligand expression, identified significant ligand-receptor pairs in triple-negative breast cancer in the cbioportal-METABRIC dataset. In total, we identified 73 significantly correlated LR-pairs (**Supplementary Table S5**).

**Figure 4:**
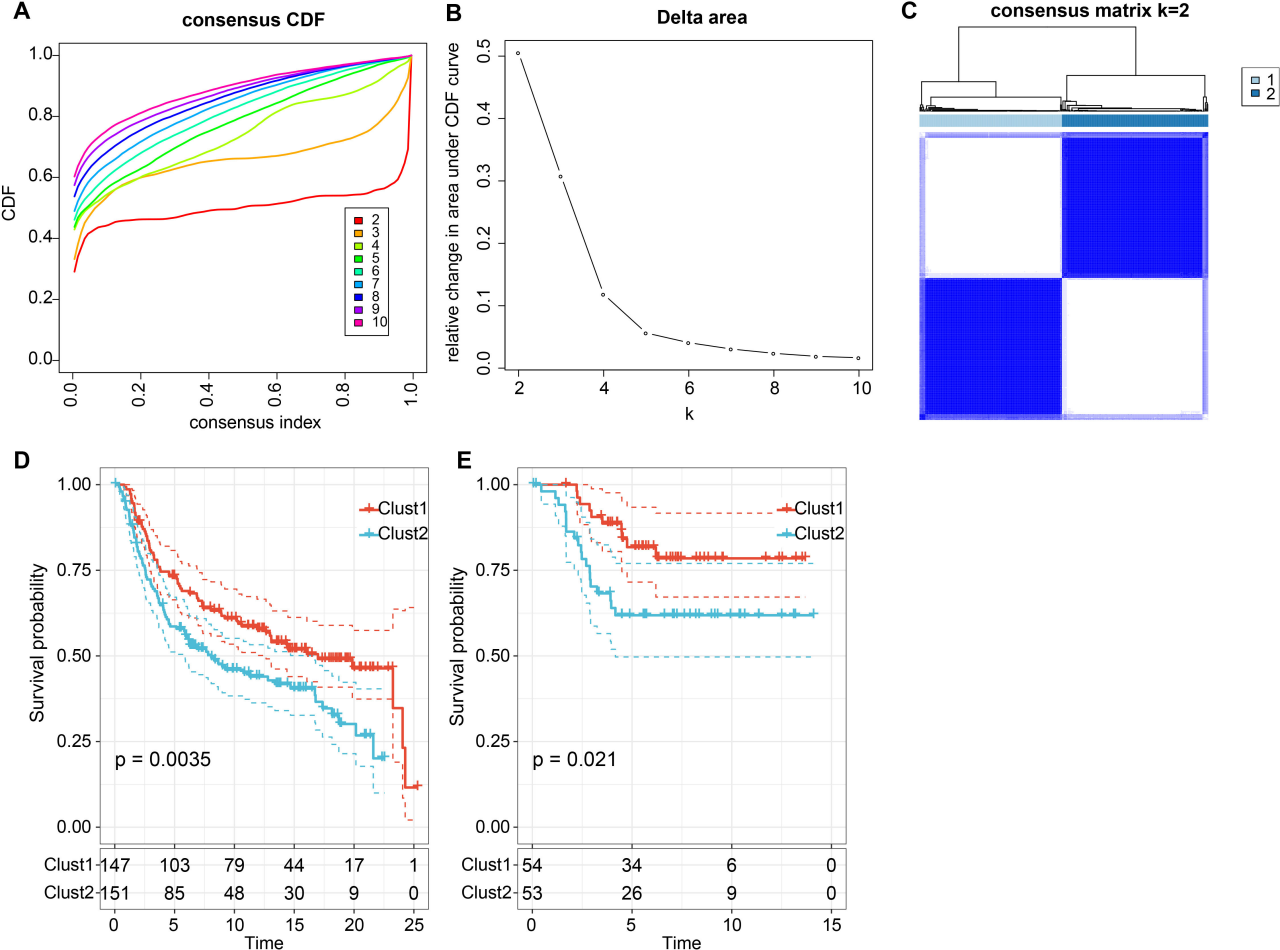
Consensus clustering based on LR pairs on TNBC. **(A)** Cumulative Distribution Function (CDF) curve of the samples in the METABRIC cohort; **(B)** Delta area curve of consensus clustering for the METABRIC cohort, indicating the relative change in the area under the CDF curve for each category number k compared with k – 1. The horizontal axis represents the category number k and the vertical axis represents the relative change in the area under the CDF curve; **(C)** Heatmap of METABRIC sample clustering when consensus k equals 2; **(D)** Overall survival (OS) curve based on LR-pairs molecular subtypes in the METABRIC cohort (*p* = 0.0035); **(E)** Overall survival (OS) curve of molecular subtypes in the GSE58812 dataset (*p* = 0.021).

Furthermore, we determined the expression strength of ligand-receptor pairs by summing the gene expression values of receptors and ligands and selected significant prognostic ligand-receptor pairs (*P* < 0.05) for molecular subtyping. We included 57 LR-pairs with substantial correlations and prognostic significance (**Supplementary Table S6**). We analyzed 298 triple-negative breast cancer samples from the cbioportal-METABRIC cohort and determined the optimal cluster number based on the cumulative distribution function (CDF) and Delta area curve (**Figures 4A, B**). Ultimately, we chose k = 2, leading to two molecular subtypes (**Figure 4C**). Further examination of these two subtypes’ prognostic characteristics revealed considerable differences in their prognosis, as illustrated in **Figure 4D**. Overall, Clust1 exhibited a more favorable prognosis, while Clust2 had a poorer prognosis. Additionally, we validated the effectiveness and robustness on the triple-negative breast cancer patient cohort from the GSE58812 dataset, observing significant differences in the prognosis of these two molecular subtypes, consistent with the training set, as shown in **Figure 4E**. These findings suggest that the two molecular subtypes based on ligand-receptor pairs are transferable across different study cohorts.

### 3.4 Genomics characteristics and clinical phenotypes of different molecular subtypes

The clinical phenotype characteristics of the two subtypes in the METABRIC cohort were analyzed and found significant differences in Grade, Pam50 classification, and CNV mutation types (**Supplementary Figures S2A–E**). Additionally, we showcased the top 10 gene mutations in the two subtypes (**Supplementary Figure S2F**). Nevertheless, in terms of genomic alterations, there is minimal difference between the two subtypes.

**Figure 5:**
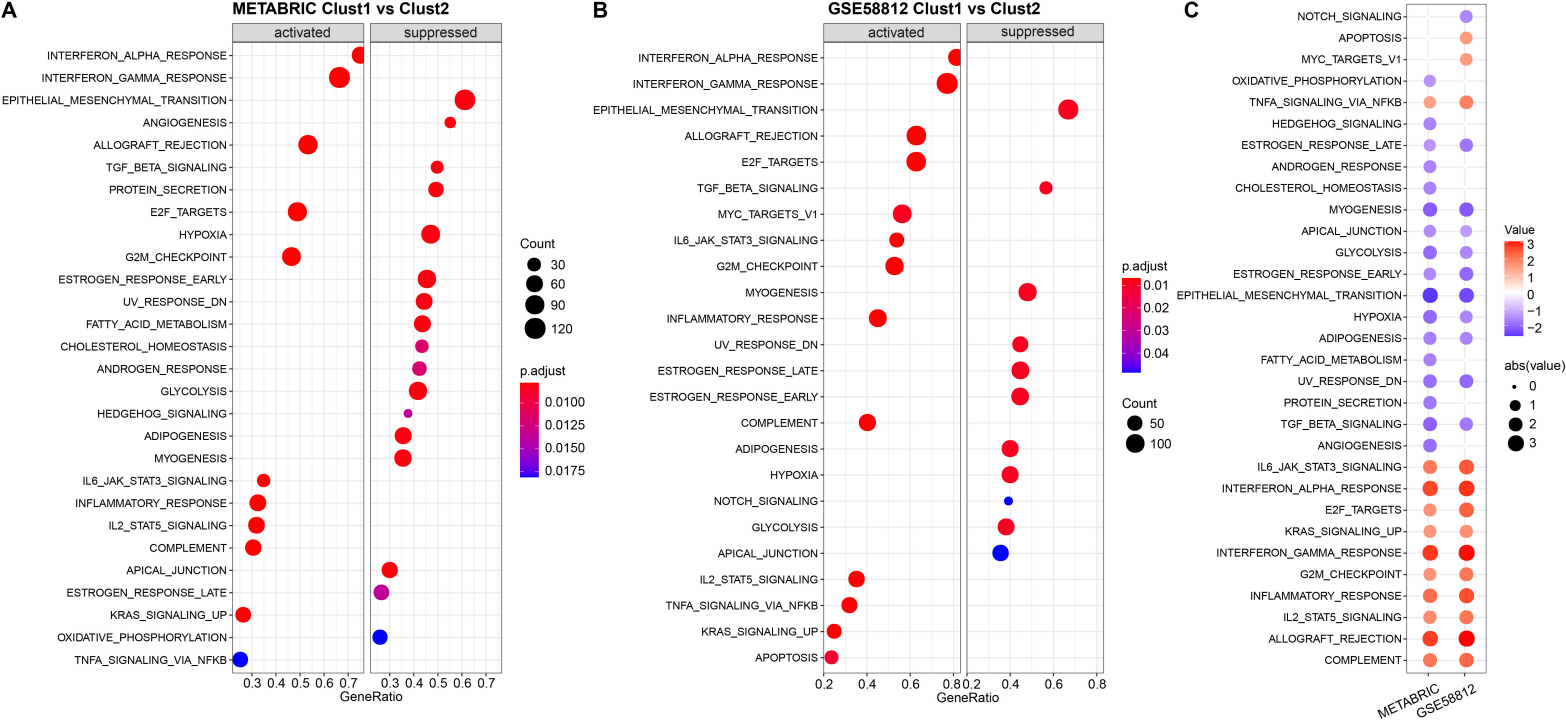
Results of Gene Set Enrichment Analysis (GSEA) for two datasets. **(A)** Bubble plot of GSEA results for the Cluster 1 vs Cluster 2 subtypes in the METABRIC cohort; **(B)** Bubble plot of GSEA results for the Cluster 1 vs Cluster 2 subtypes in the GSE58812 dataset; **(C)** Bubble plots of GSEA results across both datasets.

Next, we investigated whether there were differentially activated pathways in the different molecular subtypes. We performed gene set enrichment analysis (GSEA) using all candidate gene sets in the Hallmark database (FDR < 0.05). In the METABRIC cohort, Clust1 subtype showed significant enrichment in 28 pathways, and in the GSE58812 cohort, 24 pathways were significantly enriched (**Figures 5A, B**). Overall, pathways such as INTERFERON_GAMMA_RESPONSE, INTERFERON_GAMMA_RESPONSE were activated in both datasets, while pathways such as GLYCOLYSIS, EPITHELIAL_MESENCHYMAL_TRANSITION were suppressed in both datasets (**Figure 5C**).

**Figure 6:**
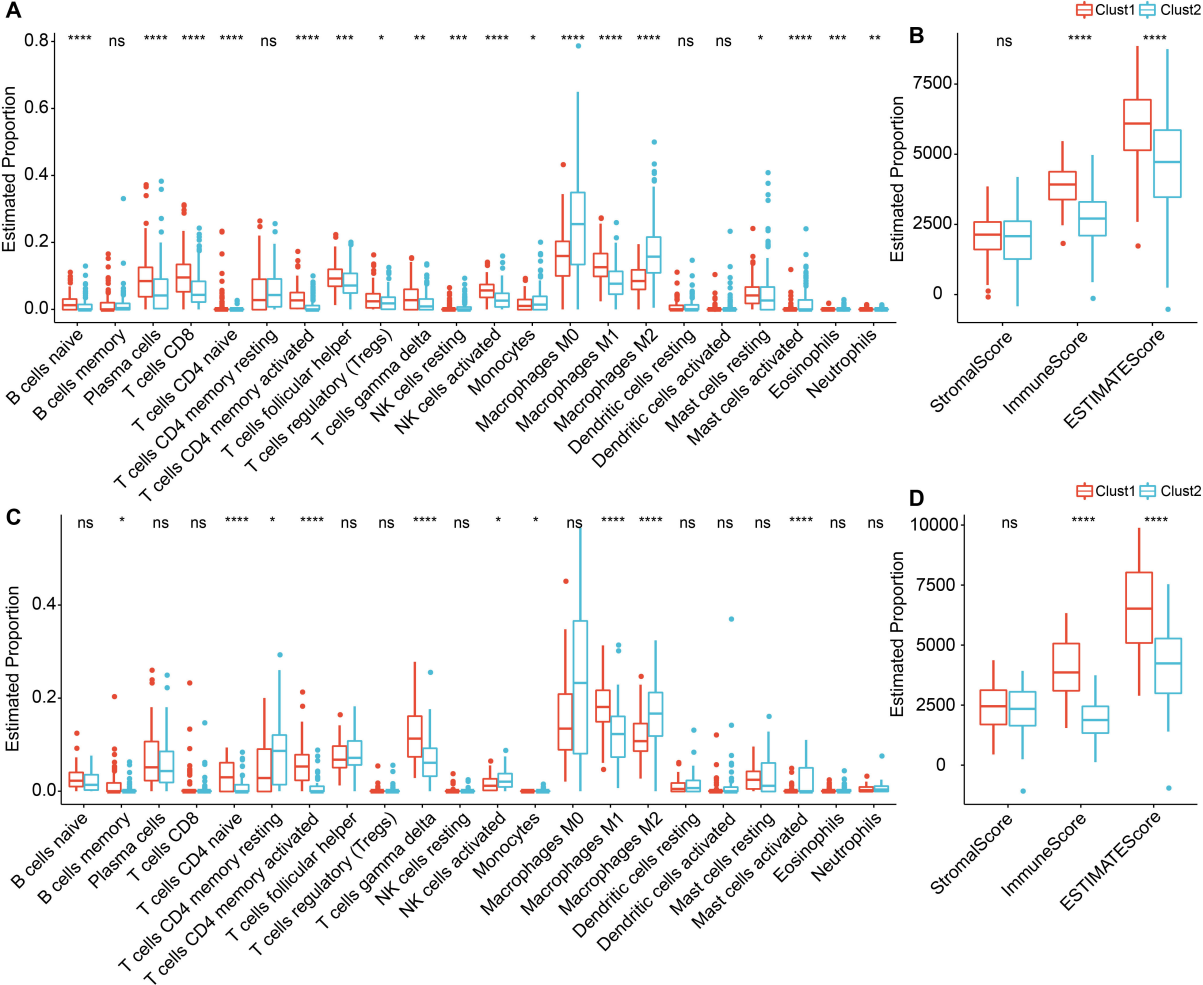
Immune infiltration levels in TNBC between Cluster 1 and Cluster 2. **(A)** Differential analysis of scores for 22 types of immune cells in the METABRIC cohort; **(B)** Differential analysis of immune infiltration levels in the METABRIC cohort; **(C)** Differential analysis of scores for 22 types of immune cells in the GSE58812 dataset; **(D)** Differential analysis of immune infiltration levels in the GSE58812 dataset. ns, *P* > 0.05; * *P* < 0.05; ** *P* < 0.01; *** *P* < 0.001; **** *P* < 0.0001. Wilcoxon rank-sum test.

To further elucidate the distinctions in the immune microenvironment of patients with ligand-receptor molecular subtypes, we employed Cibersort software to evaluate the infiltration levels of 22 immune cell types in our TNBC cohort. As depicted in **Figures 6A, C**, differences in immune cells between the distinct subtypes in the METABRIC and GSE58812 cohorts were apparent. We observed notable disparities in specific immune cells between the two molecular subtypes. For instance, T cells CD4 memory activated, T cells gamma delta, and Macrophages M1 exhibited higher scores in Clust1 than in Clust2, while Macrophages M0 and Macrophages M2 demonstrated lower scores in Clust1 compared to Clust2. In addition, we also used the ESTIMATE approach to assess immune cell infiltration, as observed, the “ImmuneScore” in clust1 subtype was higher than that in clust2 subtype in both METABRIC and GSE58812 cohorts, indicating that Clust1 subtype has relatively higher immune cell infiltration (**Figures 6B, D**).

### 3.5 Construction of a ligand-receptor pairs scoring model

**Figure 7:**
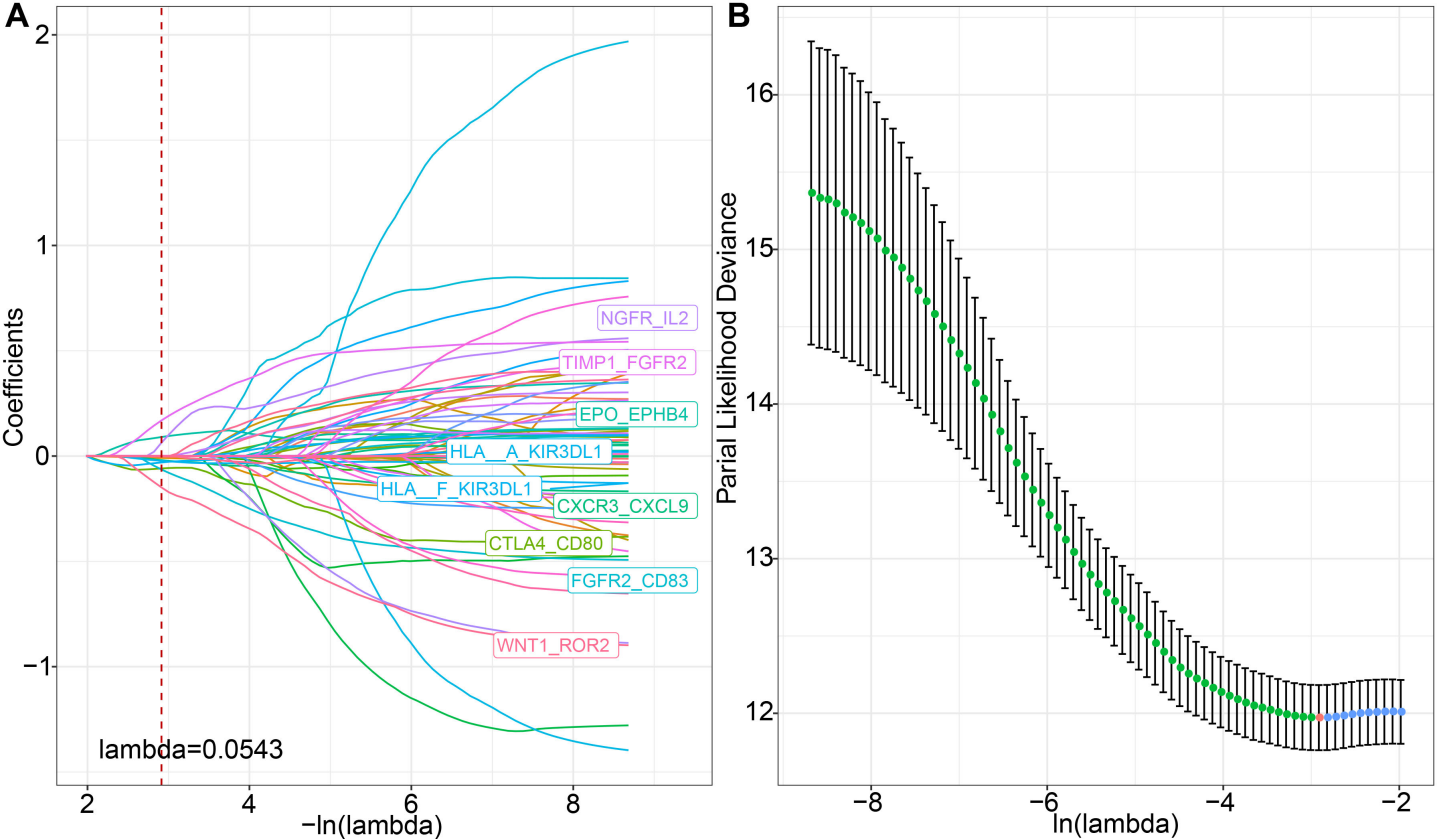
LASSO regression analysis for feature selection. **(A)** Trajectories of each predictor variable, where the x-axis represents the log value of the predictor variable lambda and the y-axis represents the coefficient of the predictor variable; **(B)** Confidence intervals for each lambda.

We discovered that molecular subtypes, based on ligand-receptor pairs, exhibit distinct pathway characteristics and varying degrees of immune infiltration. Consequently, we selected 78 prognostically significant LR pairs (*P* < 0.001) in the METABRIC cohort, employing Lasso regression to minimize the number of genes in the risk model. Initially, we examined the trajectory of each independent variable. As lambda progressively increases, the number of independent variable coefficients approaching 0 also rises (**Figures 7A, B**). We employed 10-fold cross-validation for model construction and analyzed the confidence intervals for each lambda and demonstrated that the model is optimal when lambda = 0.0543. Consequently, we selected the 9 LR pairs at lambda = 0.0543 as the target LR pairs for the subsequent step.

Furthermore, based on the 9 LR pairs obtained from the Lasso analysis, we used stepwise multivariate regression analysis, with the Akaike Information Criterion (AIC) for stepwise regression, leading to a total of six key LR pairs: WNT1_ROR2, CXCR3_CXCL9, FGFR2_CD83, TIMP1_FGFR2, NGFR_IL2, and HLA-A_KIR3DL. We then constructed an LR-pairs scoring model based on these 6 LR pairs to quantitatively analyze the LR-pairs patterns in breast cancer patients. The calculation formula for these 6 LR pairs is as follows: LR.score = -0.431 * WNT1_ROR2 - 0.081 * CXCR3_CXCL9 - 0.296 * FGFR2_CD83 + 0.467 * TIMP1_FGFR2 + 0.426 * NGFR_IL2 - 0.051 * HLA-A_KIR3DL1.

**Figure 8:**
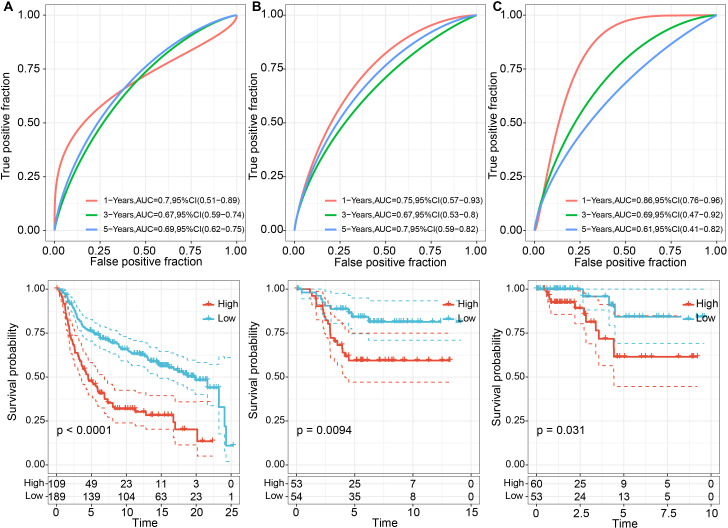
Prognostic performance of TNBC between high and low LR.scores. **(A)** ROC curves for 1, 3, and 5 years and KM curves for high and low LR.score groups in the METABRIC dataset; **(B)** ROC curves for 2, 3, and 5 years, and KM curves for high and low LR.score groups in the GSE58812 dataset; **(C)** ROC curves for 1, 3, and 5 years, and KM curves for high and low LR.score groups in the TCGA dataset.

We calculated the risk scores for each tumor sample in the METABRIC dataset based on their expression levels and performed ROC analysis for the prognostic classification of LR.score, analyzing the prognostic prediction efficiency for one (AUC = 0.7), three (AUC = 0.67), and five years (AUC = 0.69) (**Figure 8A**). Additionally, we performed a z-score transformation on the Riskscore. Samples with an LR.score greater than zero after z-score transformation were classified as high-risk, and those with a score less than zero as low-risk. as shown in **Figure 8A**, high risk score group revealed a highly significant difference in worse prognosis (*P* < 0.0001). The effectiveness of the risk model can also be transplantable, which reflected acceptable prognostic prediction in validation cohorts (1-, 3-, and 5-year OS predictions were 0.75, 0.67, and 0.70, respectively, in GSE13507; 1-, 3-, and 5-year OS predictions were 0.86, 0.69, and 0.61, respectively, in GSE32894) (**Figures 8B, C**). Across the three datasets, the risk model constructed based on the 6 LR pairs was found to be significantly correlated with prognosis, with high-risk patients having worse outcomes and low-risk patients having better outcomes. Taken together, the results suggests that the ligand-receptor pairs may contribute to the tumor microenvironment and modulate immune cell infiltration, ultimately affecting patient prognosis.

### 3.6 Correlation between LR.score and clinical variables

**Figure 9:**
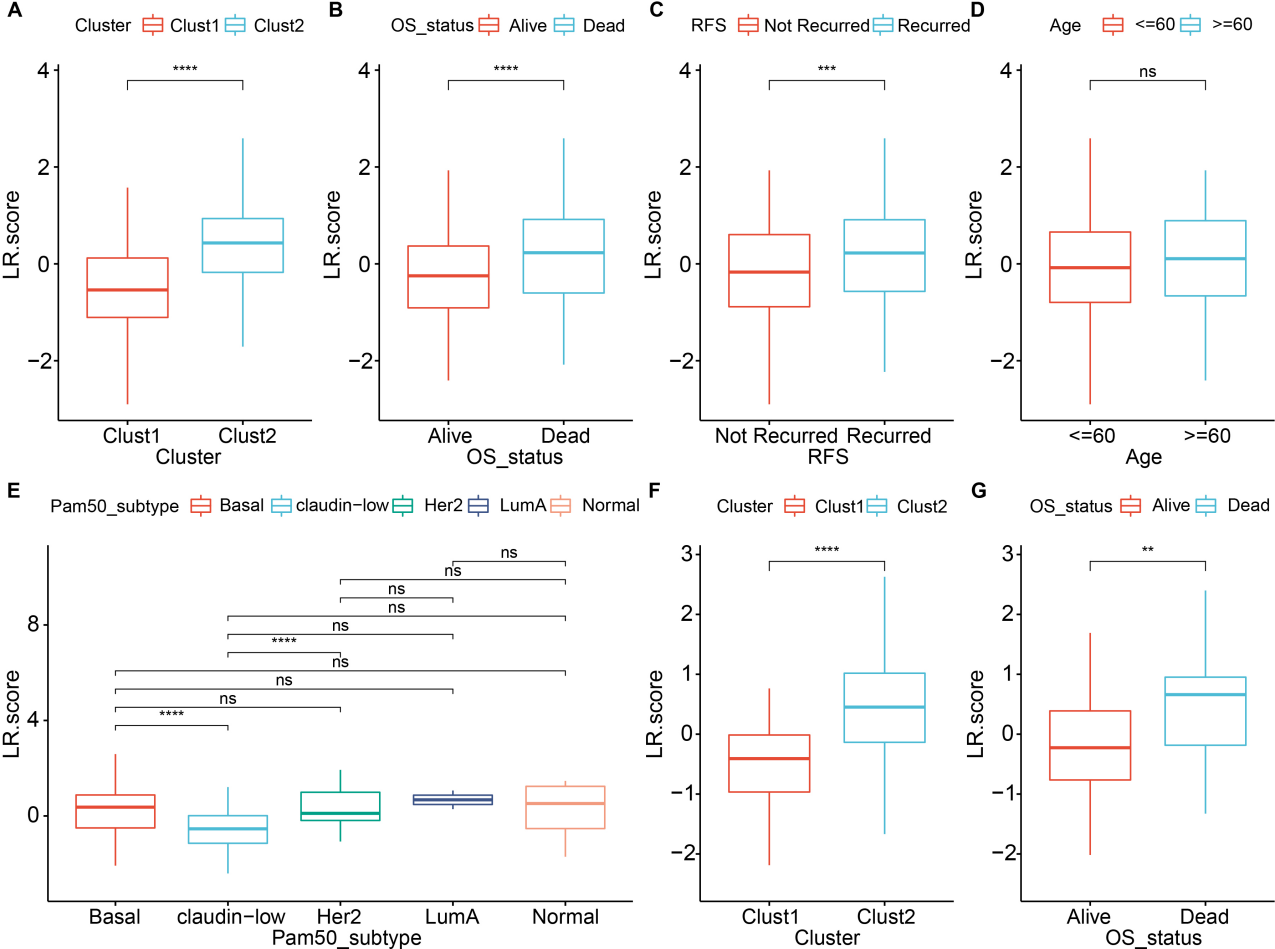
Comparison of LR score across various clinical and molecular features. **(A)** Comparison of LR scores between molecular subtypes in the METABRIC cohort; **(B)** Comparison of LR scores between different survival status in the METABRIC cohort; **(C)** Comparison of LR scores between different recurrence status in the METABRIC cohort; **(D)** Comparison of LR scores between age groups in the METABRIC cohort; **(E)** Comparison of LR scores among PAM50 molecular subtypes (Basal, Claudin-low, Her2, LumA and Normal) in the METABRIC cohort; **(F)** Comparison of LR scores between molecular subtypes in the GSE58812 dataset; **(G)** Comparison of LR scores between different survival status in the GSE58812 dataset. ns, *P* > 0.05; ** *P* < 0.01; *** *P* < 0.001; **** *P* < 0.0001. Wilcoxon rank-sum test.

Next, we compared the LR.score among various clinical features and clusters. The results showed that in the METABRIC dataset, there were significant differences in the LR.score constructed based on LR pairs for molecular subtypes, patient survival status, and recurrence status (**Figures 9A–D**, **Supplementary Table S7**). Specifically, within the Pam50 subtypes, significant differences were observed in the LR.score between Basal and Claudin-low, as well as between Claudin-low and HER2 (**Figure 9E**). In the validation cohort (GSE58812), significant differences were found in the LR.score for patient survival status and molecular subtypes constructed based on LR pairs (**Figures 9F, G**, **Supplementary Table S8**).

**Figure 10:**
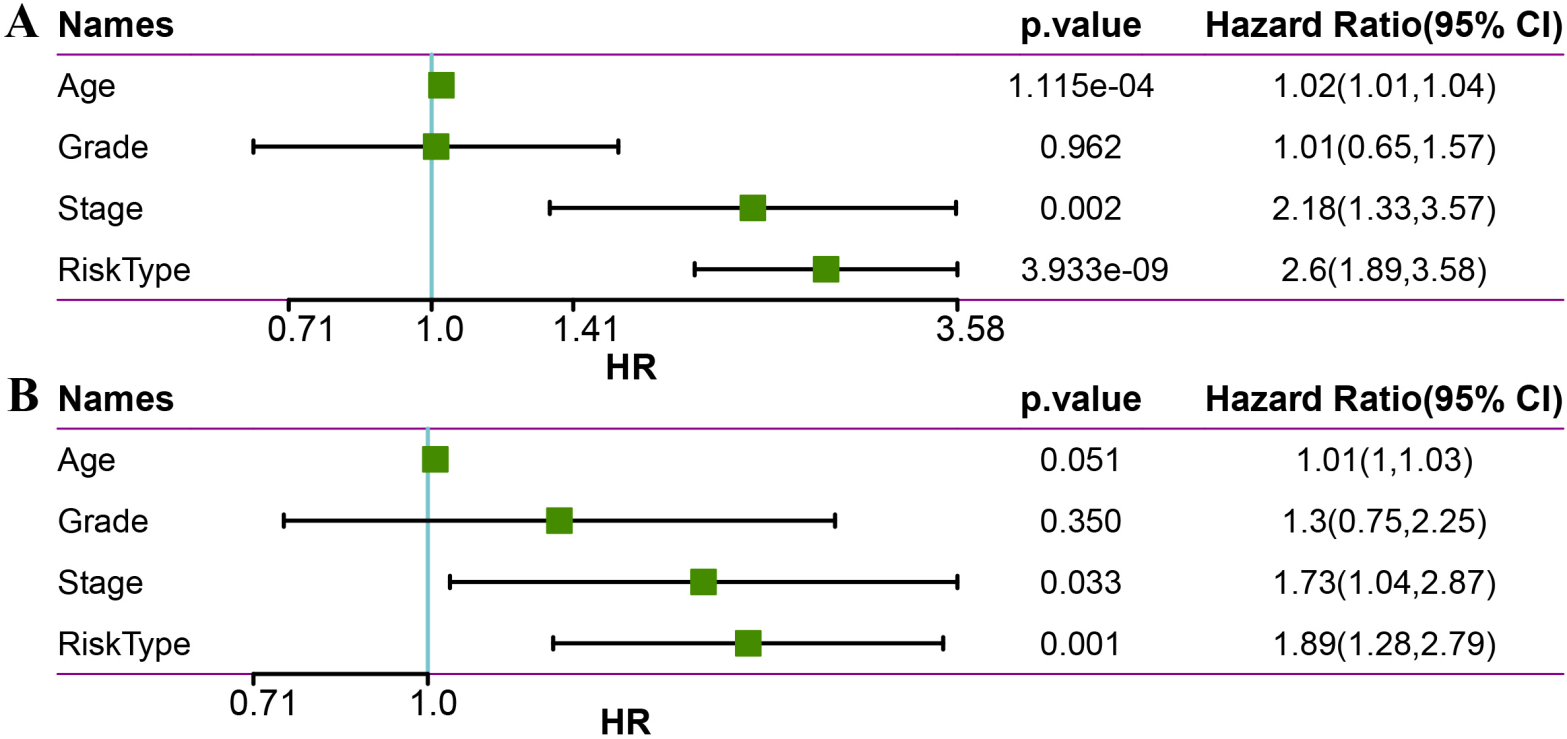
Univariate **(A)** and multivariate **(B)** analysis of age, grade, stage, and risk type in the METABRIC dataset.

To evaluate whether the LR.score can serve as an independent prognostic factor, we performed univariate Cox regression analysis and multivariate Cox regression analysis with clinical features (including Age, Stage, and Grade). The findings demonstrated that in the METABRIC dataset, univariate Cox regression analysis identified a significant association between RiskType and survival (**Figure 10A**). Likewise, multivariate Cox regression analysis showed that RiskType (HR = 1.69, 95% CI = 1.12-2.56, p <0.05) maintained a significant relationship with survival (**Figure 10B**).

**Figure 11:**
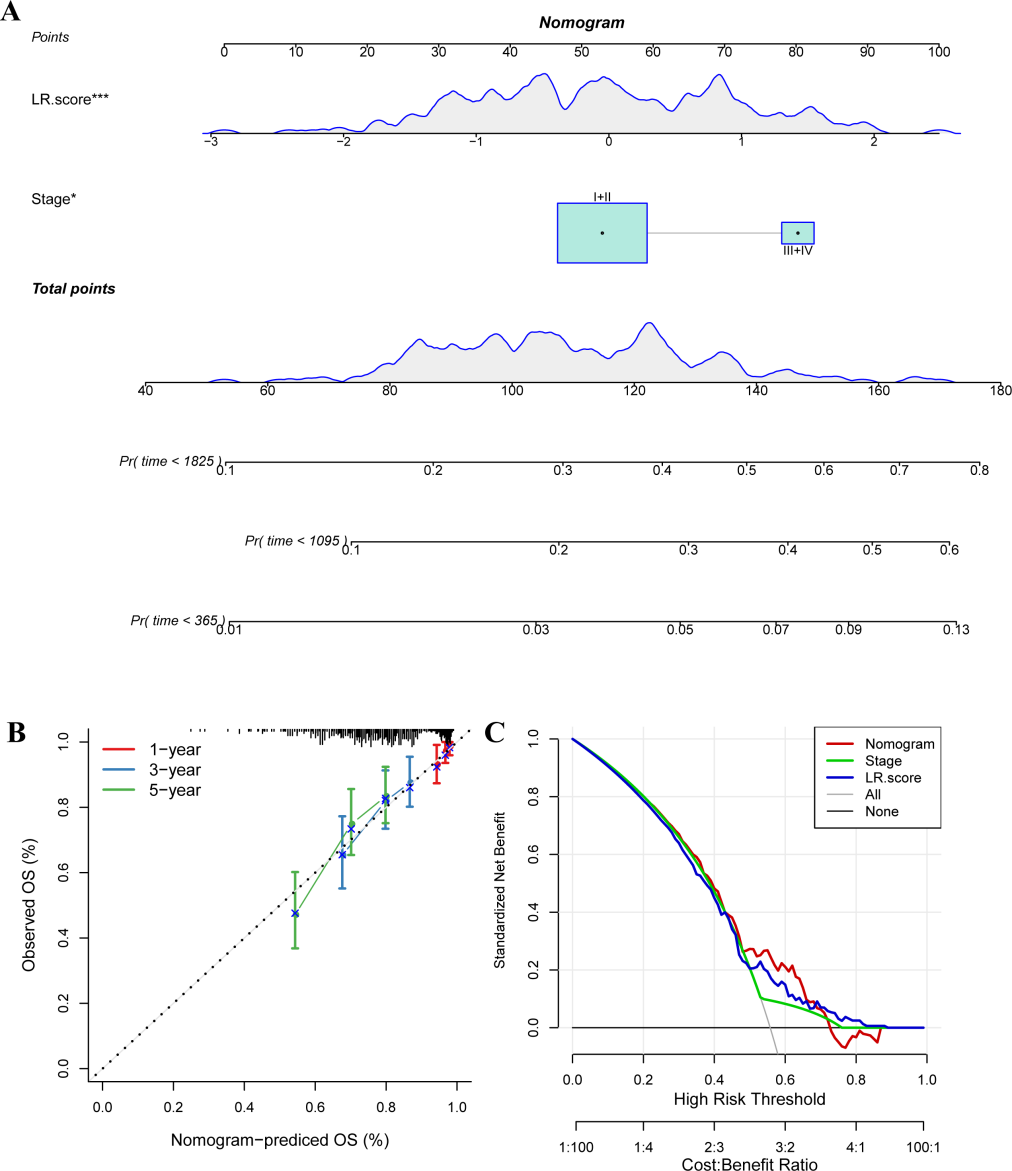
The nomogram of the LR.score and its clinical implications. **(A)** A nomogram predicting the survival probability at 1-, 3-, and 5-year intervals for TNBC patients in the METABRIC dataset. Each patient’s total score of clinical characteristics and risk score are located on the “Total points” axis, which corresponds to the survival probabilities plotted on the three axes below. **(B)** Calibration curves for the nomogram at 1-year, 3-year, and 5-year intervals. **(C)** Decision curve for the Nomogram, stage, LR.score, and all clinical factors. * *P* < 0.05; *** *P* < 0.001.

We used the METABRIC dataset to build a nomogram for the combination of Stage and LR.Score (**Figures 11A, B**). The nomogram indicated that the LR.Score has the most significant impact on survival prediction, suggesting that the 6-LR pair-based risk model can effectively predict prognosis. We plotted the Decision Curve Analysis (DCA) for Stage, Cluster, LR.Score, and Nomogram, and the results show that the Nomogram has a better performance, as shown in **Figure 11C**.

### 3.7 Correlation between LR.score and immune-related features

Furthermore, we analyzed the distribution differences of 22 immune cell scores in the LR.score groups in the training cohort (**Supplementary Figures S3A, B**). We compared the immune infiltration status in the LR.score groups and found that the StromalScore, ImmuneScore, and ESTIMATEScore in the high LR.score group were significantly lower than those in the low LR.score group (**Supplementary Figure S3C**). Moreover, we examined the relationship between LR.score and 22 immune cell scores in the METABRIC cohort and calculated the correlation between immune feature indices and immune cells using Pearson’s correlation coefficients. The results are shown in **Supplementary Figure S3D**, indicating that LR.score is significantly positively correlated with resting NK cells, M0 macrophages, and activated mast cells, while it is significantly negatively correlated with activated NK cells and M1 macrophages.

**Figure 12:**
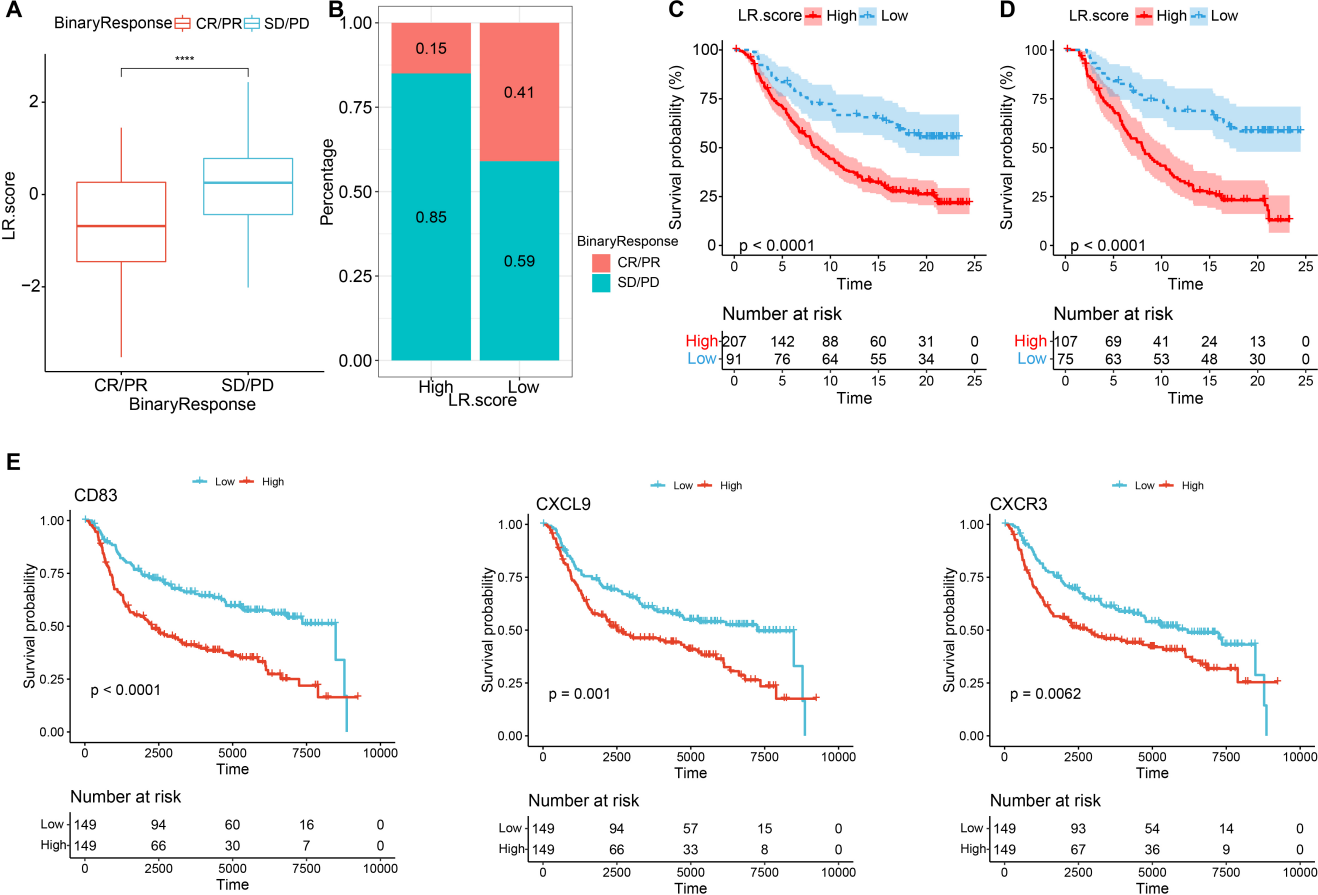
Immunotherapy performance predications based on the LR.score **(A)** Differences in the LR.score among immune therapy responses in the IMvigor210 cohort; **(B)** Distribution of immune therapy responses among different LR.score groups in the IMvigor210 cohort; **(C)** Prognostic differences between LR.score groups in the IMvigor210 cohort; **(D)** Prognostic differences between LR.score groups among early-stage patients in the IMvigor210 cohort; **(E)** Kaplan–Meier analysis of the LR genes in the METABRIC dataset, Log rank test. * *P* < 0.05; **** *P* < 0.0001.

To explore the relationship between LR.score and immunotherapy, we examined the ability of LR.score to predict patients’ responses to immune checkpoint blockade (ICB) treatment. we found that in the anti-PD1 cohort (GSE78220), CR/PR patients had better prognosis than SD/PD patients (**Figure 12A**, log-rank test, p<0.001), and SD/PD patients had lower LR.score than CR/PR response patients (**Figure 12B**). The percentage statistics performed between the low LR.score group and the high LR.score group also showed that patients in the low LR.score group had significantly better treatment outcomes (**Figures 12C, D**). These findings suggest that the LR.score may serve as a potential prognostic biomarker and predictor of immunotherapy response in TNBC patients. We used the median of LRpairs receptor gene expression as the cutoff value, and samples larger than the median were divided into the high gene expression group and samples smaller than the median were divided into the low gene expression group. Kaplan Meier curves were plotted, from which it can be seen that in the METABRIC dataset, CD83 and CXCL9-CXCR3 have significant differences in overall survival (OS) (**Figure 12E**), *P<0.05*.

### 3.8 CXCL9/CXCR3 axis silencing impairs proliferation, colony formation, and migration in MDA-MB-231 TNBC cells

**Figure 13:**
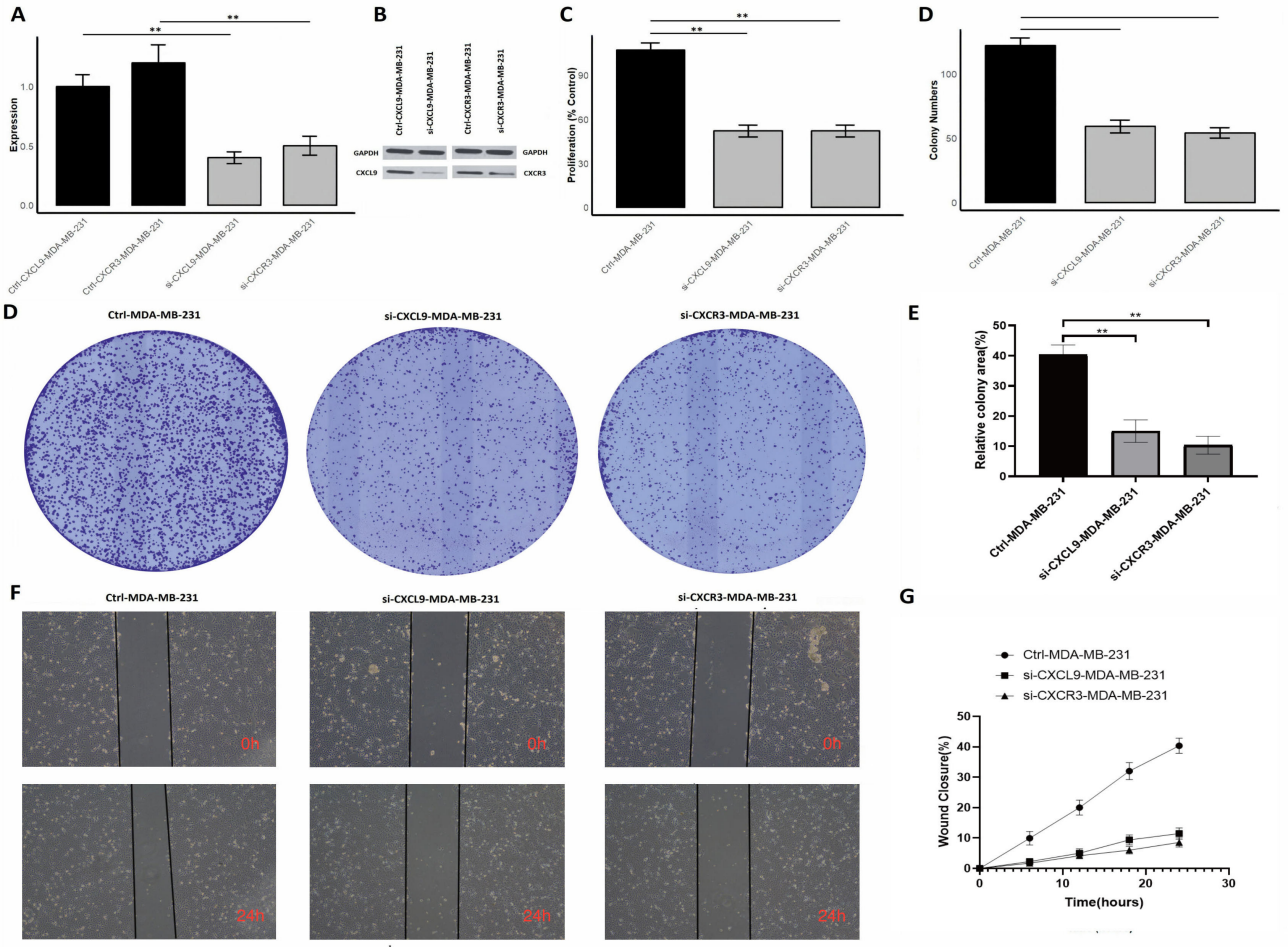
Silencing of CXCL9/CXCR3 axis reduces proliferation, colony formation, and migration in MDA-MB-231 cells. **(A)** RT-qPCR showing reduced CXCR3 and CXCL9 mRNA expression after siRNA knockdown; **(B)** Western blot confirming protein knockdown; **(C)** Proliferation assay showing decreased cell growth in siRNA-treated cells; **(D)** Colony formation assay images with fewer colonies in si-CXCR3 and si-CXCL9 cells; **(E)** Quantification of colonies showing reduced clonogenic potential; **(F)** Wound healing assay images at 0 and 24 hours showing slower wound closure in siRNA-treated cells; **(G)** Quantitative wound closure analysis showing impaired migration. p** < 0.01.

In the final part of our study, we investigated the functional roles of CXCL9/CXCR3 axis in MDA-MB-231 cells by performing gene silencing experiments using siRNA (si-CXCL9 and si-CXCR3). To assess the impact of these knockdowns, we conducted several assays, including RT-qPCR, Western blot analysis, cell proliferation, colony formation, and wound healing assays. The effective knockdown of CXCL9 and CXCR3 at the mRNA level in MDA-MB-231 cells, as measured by RT-qPCR. The expression of both CXCL9 and CXCR3 was significantly reduced in cells transfected with the respective siRNAs compared to control cells (**Figure 13A**). A marked reduction in protein expression was observed in si-CXCL9-treated and si-CXCR3 cells compared to control cells, consistent with the RT-qPCR results (**Figure 13B**, **Supplementary Figure S4**). The knockdown of CXCL9 and CXCR3 led to a significant reduction in cell proliferation rates of si-CXCL9 and si-CXCR3-treated cells compared to the control MDA-MB-231 cells (**Figure 13C**). The number of colonies formed by si-CXCL9 and si-CXCR3-treated cells was significantly lower than that of the control cells, indicating that CXCL9 and CXCR3 are critical for the clonogenic potential of MDA-MB-231 cells (**Figures 13D, E**). Images from **Figure 13F** demonstrate delayed wound closure in si-CXCL9 and si-CXCR3-treated cells compared to the control cells over a 24-hour period. Quantitative analysis in **Figure 13G** shows a significant reduction in wound closure percentage in si-CXCL9 and si-CXCR3-treated cells, further confirming the roles of these genes in promoting cell migration. Overall, these results provide strong evidence that CXCL9/CXCR3 axis play crucial roles in regulating cell proliferation, colony formation, and migration in MDA-MB-231 cells.

## 4 Discussion

The tumor microenvironment (TME) has long been acknowledged as a key determinant of cancer progression, treatment resistance, and immune escape. Prior research highlights the intricate interplay between stromal and immune cells, emphasizing the critical function of ligand–receptor (LR) interactions in shaping tumor phenotypes and driving cancer heterogeneity (17). In this study, we performed single-cell RNA sequencing (scRNA-seq) to cluster 12 subgroups and identify 10 distinct cell types, then extracted LR pairs that showed statistically significant intercellular communications. By applying these interactions to the METABRIC cohort of TNBC patients, we identified 73 LR pairs with significant correlations, among which 57 were prognostically relevant. Further analyses revealed two molecular subtypes based on LR pairs, each displaying notable differences in clinicopathological features, somatic mutation profiles, pathway activations, and immune landscapes. We evaluated the stability of the identified molecular subtypes by testing variations in key parameters such as the number of principal components and resolution parameters during preliminary analyses. The resulting subtype assignments remained largely consistent under reasonable parameter adjustments, indicating robust subtype definitions. Although a formal sensitivity analysis was not performed, these observations support the reliability of our clustering-based subtype classification. Through Lasso regression, 9 key LR genes were highlighted; subsequently, a 6-LR-pair scoring model (LR.score) was constructed via stepwise regression and multivariate analysis, underscoring the importance of these LR pairs in prognostication and therapeutic response.

Among the two molecular subtypes identified in our study, Clust1 exhibited both heightened immune infiltration and an elevated immune score, alongside prominent activation of immune-related pathways such as interferon responses. Additionally, Clust1 demonstrated a more enriched tumor microenvironment overall, as evidenced by its significantly higher ESTIMATEScore, while maintaining comparable stromal content relative to Clust2. These findings suggest that Clust1 is characterized by a more immune-active phenotype, potentially indicative of enhanced immunogenicity and immune system engagement (18). Pathway enrichment analyses further revealed that Cluster 1 exhibited prominent signatures of epithelial–mesenchymal transition (EMT), angiogenesis, and interferon response pathways (including both alpha and gamma responses), which are commonly associated with heightened immune activity, tumor invasiveness, and metastasis. Additionally, Cluster 1 showed significant activation of metabolic pathways such as fatty acid metabolism and cholesterol homeostasis, indicating distinct metabolic reprogramming. In contrast, Cluster 2 displayed relatively lower activation of these pathways, suggesting a less invasive and metabolically stable phenotype (19). Notably, there was a pronounced infiltration of activated T cells—particularly CD4 memory T cells and γδ T cells—as well as M1 macrophages, underscoring an enhanced anti-tumor immune response within this subtype (20, 21). Meanwhile, diminished levels of M0 and M2 macrophages may mitigate immunosuppressive influences, thus bolstering immune-mediated tumor control (22, 23). Taken together, these findings imply that Clust1 represents a more immunologically active phenotype, offering potentially important clues for refined patient stratification and more targeted therapeutic approaches.

A key highlight of our analysis is the involvement of the CXCL9–CXCR3 axis in shaping the immune landscape of TNBC. Chemokines such as CXCL9 are recognized for their potent capacity to recruit T lymphocytes, thereby activating and sustaining robust anti-tumor immune responses (24, 25). The receptor CXCR3 is widely expressed on activated T cells, natural killer (NK) cells, and other immune subsets, facilitating the chemotaxis of these effector cells into the tumor microenvironment (TME) (26). In multiple cancer types, elevated CXCL9/CXCR3 signaling has been associated with improved prognosis, primarily due to effective tumor-immune cell interactions that curb tumor progression (27). Within TNBC specifically, harnessing or enhancing the CXCL9–CXCR3 interaction could potentially strengthen immune infiltration and cytotoxicity, laying the groundwork for novel immunotherapeutic strategies that boost anti-tumor immunity (28). Mechanistically, CXCL9–CXCR3 signaling orchestrates multiple layers of anti−tumor immunity in TNBC. CXCL9, produced chiefly by IFN−γ–stimulated endothelial cells and M1−polarised macrophages, binds CXCR3 on activated CD^+^ T cells, Th1 cells and NK cells, triggering PI3K–AKT−dependent chemotaxis and intertumoral retention of effector lymphocytes (29). Functionally, our siRNA knockdown experiments show that disrupting this axis impairs proliferation, colony formation, and migration of MDA-MB-231 cells, indicating both autocrine and paracrine effects on tumor and immune dynamics. The LR.score model developed in this study offers a quantitative measure that integrates multiple critical ligand–receptor (LR) interactions, including CXCL9–CXCR3. In both the METABRIC and GSE58812 cohorts, patients with higher LR.score values demonstrated significantly poorer survival, highlighting the score’s robust prognostic utility (30). Moreover, univariate and multivariate Cox regression models in the METABRIC dataset confirmed that LR.score serves as an independent predictor of patient outcomes. These results imply that the cumulative effect of key LR interactions captured by the LR.score is capable of reflecting the overall degree of immune crosstalk and tumor aggressiveness, thereby providing clinicians with an additional tool for risk stratification. Furthermore, the individual components of the LR.score reflect meaningful biological processes in TNBC. For instance, CXCL9–CXCR3 and FGFR2–CD83, both with negative coefficients, are associated with enhanced immune activation and T cell recruitment, supporting their protective roles in tumor control. In contrast, TIMP1–FGFR2 and NGFR–IL2 have positive coefficients and may reflect pathways involved in tumor progression or immune suppression. These coefficients provide insight into how the balance of immune-promoting versus tumor-supporting interactions contributes to the LR.score’s prognostic relevance.

Additionally, the LR.score shows a clear association with the immune landscape of TNBC. Although we did not directly assess PD-L1 levels or predefined immune gene expression signatures, tumors with low LR.score values exhibited higher infiltration of CD^+^ T cells and M1 macrophages, as estimated by CIBERSORT and ESTIMATE. This inverse relationship suggests that LR.score may reflect the immunological activity of the tumor microenvironment and could serve as a surrogate biomarker for identifying immunologically “hot” tumors. Such a tool may be particularly valuable in clinical contexts where direct immune profiling is not feasible.

Immune checkpoint molecules—including PD-1, PD-L1, and CTLA-4—are pivotal to tumor immune evasion mechanisms, and monoclonal antibodies targeting these pathways have revolutionized cancer therapy (31–33). In our anti–PD1 cohort, the LR.score reliably distinguished between patients who are likely to derive clinical benefit from ICB therapy and those who are less responsive. Patients in the low LR.score group displayed enhanced responsiveness to checkpoint inhibitors, coupled with improved clinical outcomes. In contrast, elevated LR.score values correlated with suboptimal ICB responses and shorter survival. This distinction supports the notion that LR.score can serve as a biomarker for identifying TNBC patients who are prime candidates for immunotherapy, potentially sparing non-responders from the side effects and costs of ineffective treatments.

The implications of these findings are far-reaching. First, they emphasize the functional importance of LR-mediated crosstalk in shaping both tumor behavior and therapy response, thereby underscoring the value of systematically dissecting LR networks in diverse cancer settings. Second, our work suggests that targeting specific ligand–receptor axes—including CXCL9–CXCR3—may amplify the efficacy of existing immunotherapies by further promoting T-cell and NK-cell recruitment into the TME. Future endeavors should examine whether combining LR-focused interventions with checkpoint inhibitors can induce synergistic anti-tumor effects. Additionally, *in vitro* and *in vivo* validation of these LR interactions, along with expanded clinical trials incorporating LR.score–based stratification, would help consolidate our observations and guide the development of more tailored treatments.

Taken together, our study lays a foundation for future therapeutic approaches targeting LR interactions. The CXCL9–CXCR3 axis, in particular, may serve not only as a biomarker of immunologically active tumors but also as a therapeutic target to enhance immune infiltration and improve response to checkpoint inhibitors. Further exploration of drugs or biologics modulating this axis, or combining LR modulation with ICB, could offer promising directions for improving outcomes in TNBC and potentially other immunologically cold cancers.

## 5 Conclusion

By integrating single-cell transcriptomic data with large-scale clinical cohorts, our study demonstrates the pivotal role of ligand–receptor (LR) interactions—particularly the CXCL9–CXCR3 axis—in shaping TNBC progression and immune responsiveness. The LR.score we constructed effectively stratifies patient risk and predicts response to immunotherapy, underscoring its potential as a robust prognostic biomarker. Clinically, the LR.score could be instrumental in guiding personalized treatment strategies by identifying patients who may benefit from specific therapies, such as ICB or targeted treatments, based on their LR interaction profiles. This approach not only enhances prognostic precision but also supports the development of tailored therapeutic interventions, paving the way for more effective and individualized management of TNBC.

## Data Availability

The original contributions presented in the study are included in the article/**Supplementary Material**. Further inquiries can be directed to the corresponding author.

## Supplementary material

Supplementary Figure 1 The t-SNE plot showing the expression of marker genes.

Supplementary Figure 2
**(A)** Comparison of the two subtypes across various stages; **(B)** Comparison of the two subtypes in terms of Grade; **(C)** Comparison of the two subtypes based on the Pam50 subtypes; **(D)** Age comparison of patients across the two subtypes; **(E)** Comparison of CNV mutation types in the two subtypes; **(F)** Heatmap of the top 10 gene mutations in the two subtypes.

Supplementary Figure 3
**(A)** Distribution of 22 types of immune cell components across different LR.score groups within the METABRIC cohort. **(B)** Comparative analysis of the 22 types of immune cell components among different LR.score groups within the METABRIC cohort. **(C)** Variation in immune cell infiltration among different LR.score groups within the METABRIC cohort. **(D)** Correlation analysis between LR.score and immune cell scores.

Supplementary Figure 4 Uncropped Western Blot bands of CXCL9, CXCR3, and GAPDH in MDA-MB-231 cells.
