# Supplementary figures and images for: Single-cell ligand–receptor profiling reveals an immunotherapy-responsive subtype and prognostic signature in triple-negative breast cancer
Source: Front Immunol. 2025 Jun 10;16:1590951. doi: 10.3389/fimmu.2025.1590951 (PMC12185476; doi:10.3389/fimmu.2025.1590951)

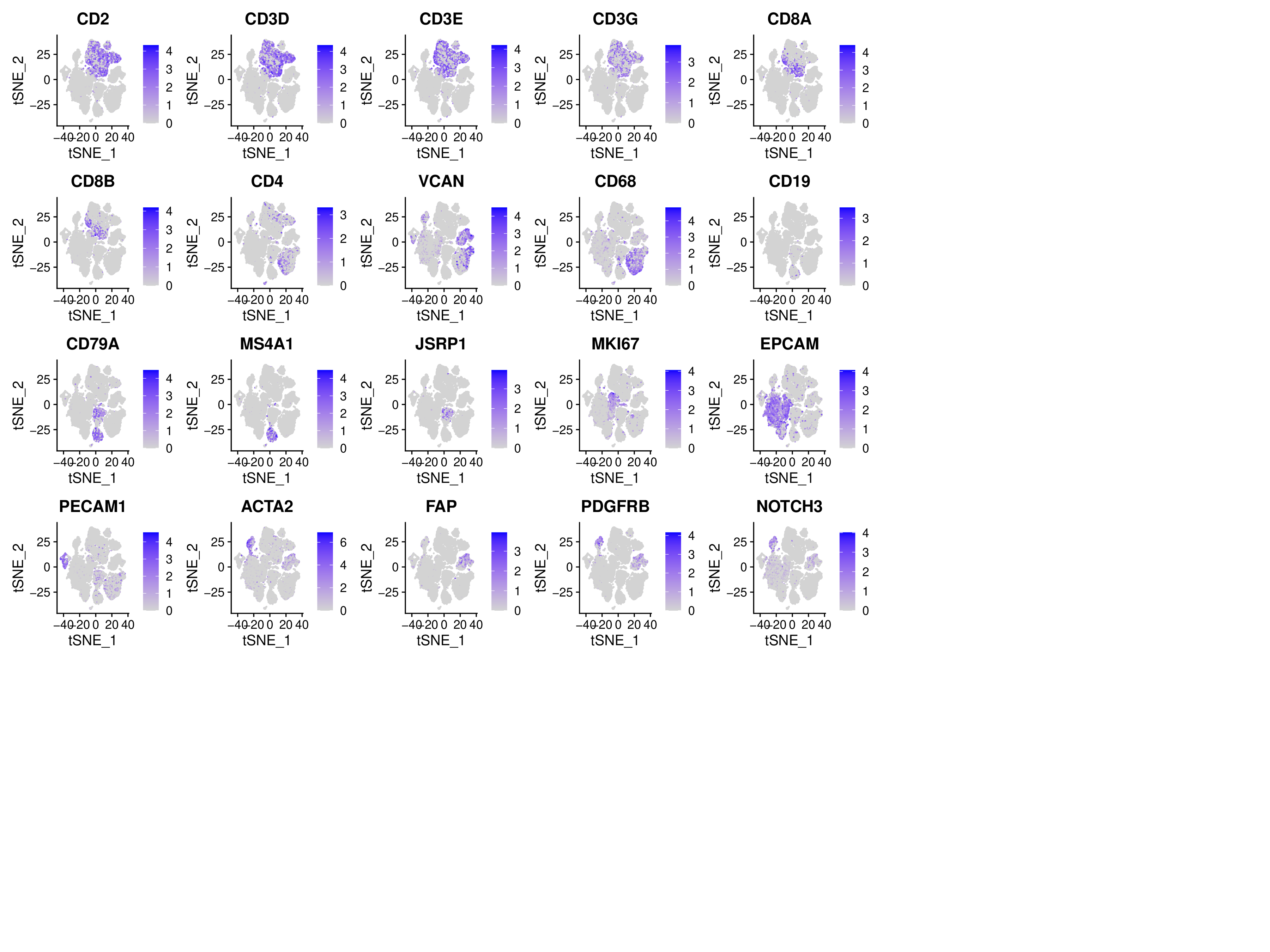

Supplement: Supplementary Figure 1 — The t-SNE plot showing the expression of marker genes. [file Image1.jpeg]

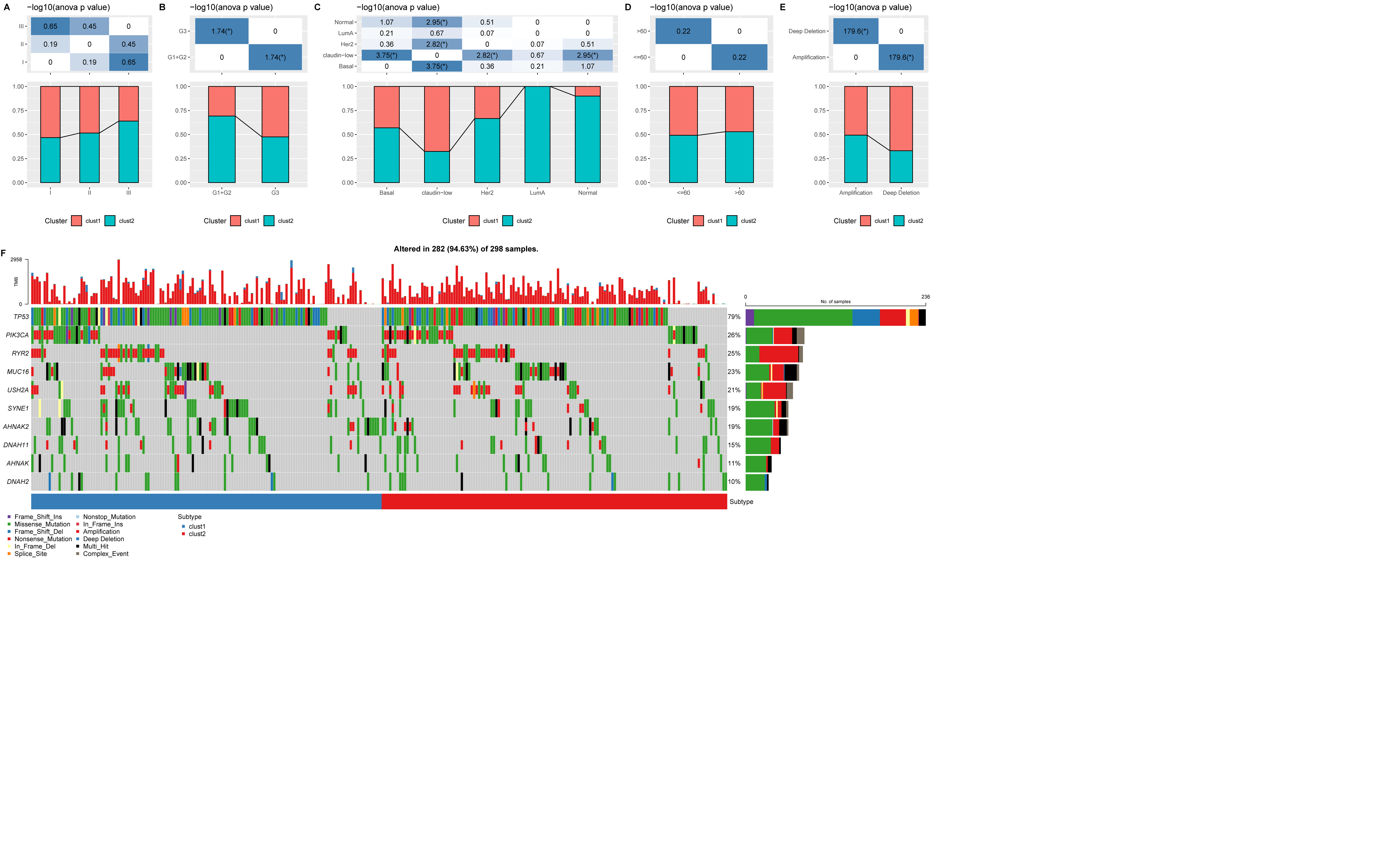

Supplement: Supplementary Figure 2 — (A) Comparison of the two subtypes across various stages; (B) Comparison of the two subtypes in terms of Grade; (C) Comparison of the two subtypes based on the Pam50 subtypes; (D) Age comparison of patients across the two subtypes; (E) Comparison of CNV mutation types in the two subtypes; (F) Heatmap of the top 10 gene mutations in the two subtypes. [file Image2.jpeg]

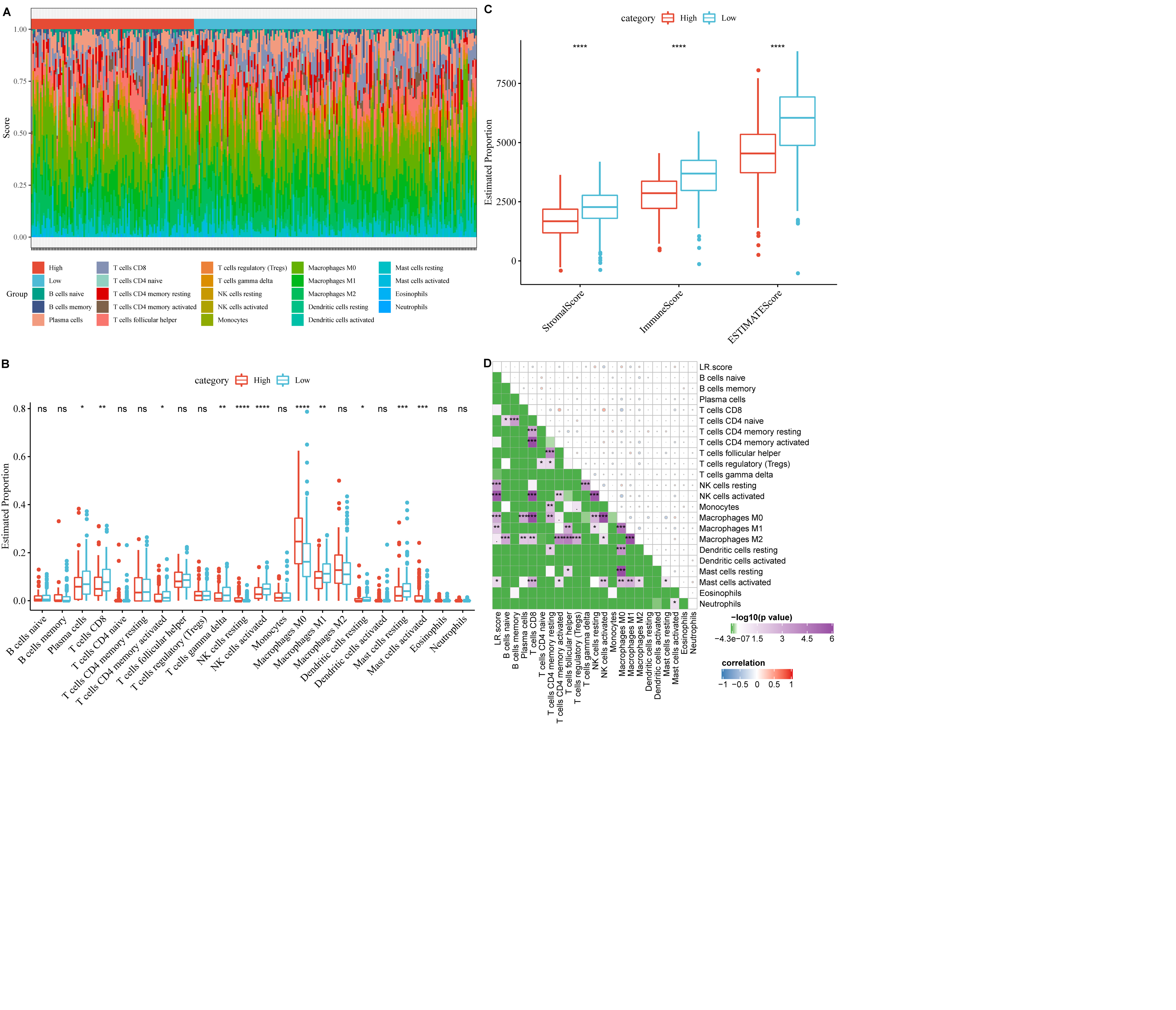

Supplement: Supplementary Figure 3 — (A) Distribution of 22 types of immune cell components across different LR.score groups within the METABRIC cohort. (B) Comparative analysis of the 22 types of immune cell components among different LR.score groups within the METABRIC cohort. (C) Variation in immune cell infiltration among different LR.score groups within the METABRIC cohort. (D) Correlation analysis between LR.score and immune cell scores. [file Image3.jpeg]

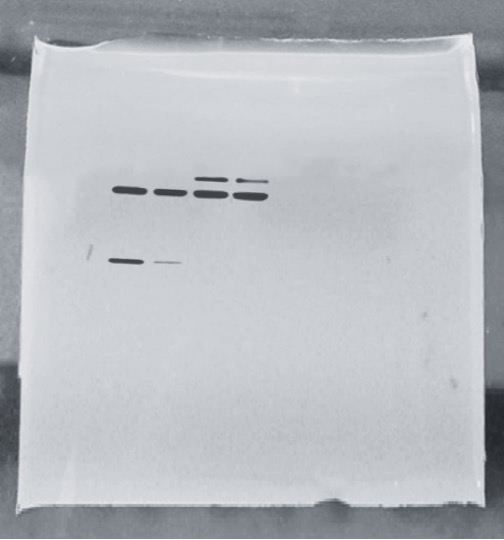

Supplement: Supplementary Figure 4 — Uncropped Western Blot bands of CXCL9, CXCR3, and GAPDH in MDA-MB-231 cells. [file Image4.jpeg]
