# Supplementary material for: Single-cell ligand–receptor profiling reveals an immunotherapy-responsive subtype and prognostic signature in triple-negative breast cancer
Source: Front Immunol. 2025 Jun 10;16:1590951. doi: 10.3389/fimmu.2025.1590951 (PMC12185476; doi:10.3389/fimmu.2025.1590951)
Supplement: Supplementary file 13 [file Table9.docx]

Raw data:

https://drive.google.com/drive/folders/15MqEhKDB0cv3OHxzgox0XIJSHapHsNMc?usp=sharing
